# Uncovering the Burden of Diabetes in Ekiti State, Nigeria: Insights From a Statewide, Household-Based, Cross-Sectional Study

**DOI:** 10.7759/cureus.50686

**Published:** 2023-12-17

**Authors:** Kehinde S Oluwadiya, Taiwo H Raimi, Samuel A Dada, Bolade F Dele-Ojo, Adekunle O Adeoti, Oluremi O Solomon, Eyitope Amu, Jacob O Awoleke, Samuel A Atiba, Adefunke O Babatola, Mobolaji U Dada, Olumuyiwa E Ariyo, Adetunji J Omotayo, Ademola O Adelekan, Esu S Ezeani, Laofe Ogundipe, Adebowale F Akinwunmi, Felix O Aina, Segun M Agboola

**Affiliations:** 1 Department of Surgery, Ekiti State University, Ado Ekiti, NGA; 2 Department of Medicine, Ekiti State University, Ado Ekiti, NGA; 3 Department of Community Medicine, Ekiti State University, Ado Ekiti, NGA; 4 Department of Obstetrics and Gynaecology, Ekiti State University, Ado Ekiti, NGA; 5 Department of Chemical Pathology, Ekiti State University, Ado Ekiti, NGA; 6 Department of Pediatrics, Ekiti State University, Ado Ekiti, NGA; 7 Department of Psychiatry and Behavioral Sciences, Ekiti State University, Ado Ekiti, NGA; 8 Department of Medicine, Federal Teaching Hospital, Ido Ekiti, NGA; 9 Department of Anatomic Pathology, Ekiti State University, Ado Ekiti, NGA; 10 Department of Chemical Pathology, Federal Teaching Hospital, Ido Ekiti, NGA; 11 Department of Epidemiology and Biostatistics, Ministry of Health and Human Services, Ekiti State, Ado Ekiti, NGA; 12 Department of Community Medicine and Psychiatry, Afe Babalola University, Ado Ekiti, NGA; 13 Department of Family Medicine, Ekiti State University, Ado Ekiti, NGA; 14 Department of Family Medicine, Afe Babalola University, Ado Ekiti, NGA

**Keywords:** nigeria, ekiti state, predictors, risk factors, prevalence, diabetes mellitus

## Abstract

Introduction: Diabetes mellitus (DM) is an important global public health challenge, and the burden of the disease is huge, particularly in low- and middle-income countries (LMICs), where the majority of people with this condition reside. Undiagnosed DM is more prevalent in LMICs. The aim of this study is to determine the prevalence and associated factors for DM in Ekiti State.

Materials and methods: A cross-sectional, household-based survey using a four-stage multistage sampling design and the World Health Organization (WHO)-STEPS survey manual was conducted from July to September 2020 as a part of the Ekiti State coronavirus disease 2019 (COVID-19) survey. Of the 5,145 sampled households, 4,726 individuals gave consent to participate in the survey. Out of these, 3043 had fasting plasma glucose results available and were included in the analysis.

Results: There were 2257 (74.2%) women and 786 (25.8%) men. The prevalence of DM was 6.5% (6.5% in males and 6.6% in females, P = 0.946). Diabetes was found to be more prevalent among those with a secondary school education or higher (10.9%); employed in the formal sector (13.4%); separated, divorced, or widowed (8.5%); with raised blood pressure (9.3%); and who were aged 30-59 years (all P < 0.05). Multivariable logistic regression showed that age, education, occupation, and hypertension were all positively and significantly associated with an increased risk of DM.

Conclusion: The prevalence of DM in Ekiti State is high, and its predictors include advancing age, hypertension, education, and occupation. This calls for scaling up public health interventions for controlling DM, targeting the identified risk factors among the people of Ekiti.

## Introduction

Diabetes mellitus (DM) is a significant public health issue, and it has significant financial consequences [1]. The burden of DM is rising globally, including in developing nations like Nigeria. In 2021, it was estimated that 536 million people had DM worldwide; by 2045, this number is expected to rise to 783 million [1]. Diabetes caused at least USD 966 billion in health expenditure [1].

According to a systematic review by Uloko et al., 5.8% of Nigerians had DM [2]. The review did not include data from Ekiti State because no study had been conducted in the state as of that time. A statewide study in Ogun State, south-west Nigeria, determined the prevalence of diabetes to be 5.1% [3]. The majority of people with diabetes mellitus live in developing or low- and middle-income countries (LMIC), and most of them are undiagnosed [1]. Factors associated with DM include age, sex, marital status, income, education, obesity, history of hypertension, and family history of diabetes, alcohol, and smoking [4,5].

In spite of the public health significance of DM, its prevalence and predictors in Ekiti State are unknown because no study that employed a representative sample of the state has been conducted. Previous reports were derived from hospital records and a few communities [6,7]. It is therefore necessary to determine the prevalence and determinants of DM in Ekiti State. This will promote the development of evidence-based policy by the government and other relevant stakeholders. This study, therefore, aims to determine the prevalence and associated factors for DM in Ekiti State.

## Materials and methods

Study area

The study was conducted in Ekiti State, which comprises 16 local governments, 176 wards, 2,457 settlements, and a population of 3,270,798 as of 2016 [8]. The Ekitis form one of the largest ethnic groups in Yorubaland. Ekitis are culturally homogeneous and speak a dialect of the Yoruba language known as Ekiti. The homogeneous nature of Ekiti confers on the state some uniqueness among the states of the federation. An important feature of the state is the large number of hills it possesses, from which it derives its name. And a typical Ekiti man cannot do without "Iyan" (pounded yam) in a day, with melon soup, crowned with bush meat [9].

Study design

We conducted a household-based, descriptive cross-sectional, survey. It was a non-communicable disease (NCD) study conducted between July and September, 2020 as part of the Ekiti State COVID-19 survey. Details of the protocol have been described elsewhere [10].

Study population

The study population consisted of adult male and female residents of Ekiti who were 18 years or older.

Inclusion and exclusion criteria

Inclusion criteria were self-reported age of 18 years or older and willingness to provide informed consent. Participants were excluded from the study if they were ill, unwilling to provide consent, or if obtaining samples for testing would be difficult or impossible. Pregnant women were also excluded.

Sample size determination

The sample size estimation was based on the World Health Organization's (WHO) STEPS Manual and its accompanying Microsoft Excel spreadsheet [11]. The methodology takes into account factors such as the number of age groups and gender estimates, the design effect, and the estimated non-response. Based on this calculation, the estimated sample size was 4321.8, which was rounded up to 4322.

Sampling technique

A four-stage multistage sampling technique was used for the study. All 16 local government areas (LGAs) in Ekiti State were included in the sample. The first stage of sampling involved randomly selecting 50% of the wards within each LGA, with the urban/rural distribution of the selected wards proportional to their population distribution in the 2006 census.

Following the first stage of sampling, a list of all settlements within the selected wards was compiled, and three settlements were randomly selected from each ward using a list of computer-generated random numbers. In the third stage, 4322 households were selected by simple random sampling for the enumeration. Finally, one individual from each selected household was randomly picked for enrollment by balloting in the survey for the fourth stage.

Advocacy/community entry

Before the survey teams began their fieldwork in each selected ward or settlement, we conducted community-level mobilization. Community mobilization teams visited each settlement one to two weeks prior to the initiation of fieldwork, working with community health workers to meet key gatekeepers in the communities. The community leaders were consulted and provided with information about the purpose of the survey to share with their community members. In addition, for each settlement, targeted radio announcements were made one to two days prior to the visit to the settlement, and town criers in the community were commissioned to announce the event on the day before and on the morning of the survey.

Informed consent

Written informed consent was obtained from the participants. This was presented in both Yoruba (the local language) and English. This included all elements of informed consent required by the National Health Research Ethics Committee (NHREC) code of conduct in order for the participant to make an informed decision about whether or not to participate. Verbal consent was obtained from illiterate individuals because electronic devices were used for data collection.

Ethical consideration

Ethical approval for this study was obtained from the Ethical Committee of Ekiti State University Teaching Hospital, Ado Ekiti, Nigeria (EKSUTH/A67/2020/07/002). Informed consent forms were signed by all the study participants, and to ensure confidentiality, the data were documented without any personal identifiers.

Data collection instrument and method

A pre-tested semi-structured interviewer-administered questionnaire, adapted from the WHO manual on chronic non-communicable diseases was used for data collection. The questionnaire was collected on Android smartphones using the Open Data Kit (ODK). It elicited information about the demographic characteristics, history of awareness, previous diagnosis and treatment of diabetes among the respondents.

Anthropometric and Blood Pressure Measurements

Anthropometric parameters and blood pressure were determined using standard protocols. Participants' weight (in kilograms) and height (in meters) were measured using bathroom scales and stadiometers, respectively. Body Mass Index (BMI) was calculated as weight divided by the square of height. Blood pressure was measured twice using a digital sphygmomanometer (Omron®), and the average of the two readings was used.

Sample collection

Trained phlebotomists and laboratory technicians followed standard aseptic procedures for collecting venous blood samples. A 3-milliliter venous blood sample was collected and placed in a fluoride-oxalate specimen bottle. Samples were collected after 10-14 hours of overnight fasting, at a designated central location, such as a primary health center or meeting hall, in each local government area.

Specimen transportation, storage, and laboratory analysis

In order to prevent hemolysis, samples in each of the specimen bottles were placed in racks of fifty each and left undisturbed. The samples were transported to the Chemical Pathology Laboratory of Ekiti State University Teaching Hospital (EKSUTH) on the same day after the fieldwork. The samples were centrifuged at 3000 rpm for five minutes, and the plasma was separated and analyzed for glucose within 24 hours. Plasma glucose was determined using the glucose oxidase method [12], with a ready-to-use commercially available kit (Randox Laboratories, UK, BT29 4QY).

Definition of terms

In this study, diabetes mellitus (DM) was defined as either a self-reported previous diagnosis by healthcare professionals or a fasting plasma glucose (FPG) level of ≥7.0 mmol/L. Obesity was defined as BMI ≥30.0 kg/m^2^. Other BMI categories are underweight (≤18.5 kg/m^2^), normal (18.5-24.9 kg/m^2^), and overweight (25.0-29.9 kg/m^2^). Hypertension was defined as a BP of ≥140/90 mmHg or current use of anti-hypertensive medication.

Data analysis

Univariate analysis was presented as percentages. For bivariate analysis, continuous variables were analyzed using Student's t-test and presented as mean (standard deviation), while categorical variables were analyzed using Pearson's chi-square test and presented as frequency (percentage). A multivariate stepwise logistic regression model was used to identify the risk factors for DM, and the adjusted odds ratios with 95% confidence intervals were calculated. Statistical significance was considered at a p-value less than 0.05. Data analyses were performed using IBM SPSS version 25 for Windows (IBM Corp., Armonk, NY).

## Results

Socio-demographic characteristics of the respondents

Of the 5,145 sampled households, 4,726 (91.9%) individuals gave consent to participate in the survey. Out of those who were interviewed, 3043 (64.4%) had fasting plasma glucose results available for analysis and were included in the analysis. The mean age was 44.4 ± 18.4 years, and the participants were made up of 2257 (74.2%) women and 786 (25.8%) men. Most of the participants were between the ages of 18 and 59 years (76.2%), currently married (85.6%), and self-employed (68.0%). The majority of the participants lived in rural areas (59.3%), had at least a secondary education (57.6%), had a low level of physical activity (95.9%), and did not smoke or drink alcohol at the time of the survey. The socio-demographic characteristics of the participants are shown in Table 1. The general steady decline in percentages of participants as the age range increases was similar to the last census report for Ekiti State.

**Table 1:** General characteristics and prevalence of diabetes mellitus among the participants.

Variables	N (%)	Diabetes prevalence N (%)	χ^2^ (p-values)
Total	3043	199 (6.5)	
Gender			
Male	786 (25.8)	51 (6.5)	0.946
Female	2257 (74.2)	148 (6.6)	
Age group (years)			
18–29	666 (21.9)	11 (1.7)	<0.001
30–44	973 (32.0)	100 (10.3)	
45–59	680 (22.3)	42 (6.2)	
60–69	373 (12.3)	31 (1.0)	
70+	351 (11.5)	15 (0.5)	
Residential areas			
Rural	1804 (59.3)	101 (5.6)	0.11
Urban	1239 (40.7)	98 (7.9)	
Education			
No formal education/less than primary school completed	569 (18.7)	27 (4.7)	<0.001
Primary school completed	791 (27.1)	49 (6.2)	
Secondary school completed	1143 (39.1)	64 (5.6)	
Above secondary school completed	540 (18.5)	59 (10.9)	
Marital status			
Single	379 (12.5)	12 (3.2)	0.016
Currently married	2603 (85.6)	182 (7.0)	
Separated/divorced/widowed	59 (1.9)	5 (8.5)	
Employment/occupation			
Employed in formal sector	366 (12.0)	49 (13.4)	<0.001
Self-employed	2070 (68.0)	118 (5.7)	
Unemployed	607 (19.9)	32 (5.3)	
Level of physical activity			
Low	2919 (95.9)	196 (6.7)	0.152
Medium	108 (3.5)	3 (2.8)	
High	16 (0.5)	0 (0.0)	
Smoking			
Not currently smoking	2972 (97.7)	194 (6.5)	0.862
Currently smoking	71 (2.3)	5 (7.0)	
Alcohol drinking			
No	2596 (85.3)	168 (5.5)	0.714
Yes	447 (14.7)	31 (6.9)	
Hypertension			
Present	581 (21.1)	54 (9.3)	0.003
Not present	2174 (78.9)	128 (5.9)	
Body Mass Index			
Underweight	224 (8.0)	12 (5.4)	0.545
Normal weight	1397 (50.1)	97 (6.9)	
Overweight	736 (26.4)	41 (5.6)	
Obese	431 (15.5)	30 (7.0)	

Prevalence of diabetes mellitus

One hundred and ninety-nine (6.5%) of the study participants had DM, defined as FPG ≥7.0 mmol/L and/or a self-reported diagnosis of diabetes. There was no significant difference between males and females (6.5% for males and 6.6% for females, P = 0.946) or between participants in rural and urban settlements (5.6% for rural and 7.9% for urban, P = 0.11). However, diabetes was found to be more prevalent among participants aged 30-59 years, those with post-secondary school education (10.9%), who worked in the formal sector (13.4%), and were separated/divorced/widowed (8.5%) (all P < 0.05). DM was also significantly associated with the presence of raised blood pressure (9.3% versus 5.9%, P = 0.003) (Table 1).

Predictors of diabetes mellitus in Ekiti State

The results from univariate and multivariate risk factor analyses are presented in Table 2. Univariate analysis showed that age, marital status, education, occupation, place of residence, and hypertension were predictors of diabetes. In the multivariable logistic regression, however, only age, education, occupation, and hypertension (hypertension: OR = 2.283, 95% CI = 1.470-3.546) predict DM with statistical significance.

**Table 2:** Factors related to the prevalence of diabetes in the logistic regression models. CI: confidence interval; BMI: Body Mass Index; **For multivariate analysis, stepwise logistic regression was used with a 0.10 significance level for removal from the model and a significance level of 0.05 for addition to the model.

	Univariate analysis		Multivariate analysis	
Variable	Odds ratio (95% CI)	P-value	Adjusted odds ratio (95% CI)	P-value
Gender (ref: male)				
	1.011 (0.728–1.406)	0.946	-	-
Age (ref:18-29 years)			-	-
30-44	6.821 (3.630-12.817)	0.000	5.777 (2.795-11.939)	0.000
45-59	3.920 (2.000-7.681)	0.000	1.953 (0.850-4.491)	0.115
60-69	5.397 (2.680-10.871)	0.000	3.640 (1.529-8.662)	0.003
=>70	2.658 (1.208-5.852)	0.015	1.800 (0.659-4.912)	0.251
Marital status (ref: married)			-	-
Never married	0.419 (0.231-0.732)	0.004	0.680 (0.317-1.454)	0.320
Separated/divorced/widowed	0.826 (0.549-1.243)	0.359	1.258 (0.773-2.046)	0.355
Level of education (ref: less than primary school)				
Primary school completed	1.321 (0.815-2.140)	0.258	1.516 (0.855-1.688)	0.154
Secondary school completed	1.186 (0.748-1.882)	0.468	1.641 (0.900-2.990)	0.106
Above secondary school	2.453 (1.531-3.932)	0.000	2.107 (1.114-3.988)	0.022
Occupation (ref: self-employed)				
Mental work or employment in formal sector	2.557 (1.795-3.642)	0.000	1.997 (1.313-3.037)	0.001
Unemployed	0.921 (0.616-1.376)	0.686	1.392 (0.889-2.179)	0.148
Type of settlement (ref: rural)				
Urban	1.448 (1.086-1.931)	0.012	1.294 (0.945-1.770)	0.108
Currently smoking (ref: non-smoker)				
Current smokers	1.177 (0.642–2.160)	0.598	-	-
Current alcohol drinkers (ref: non-drinkers)				
Current alcohol drinkers	1.077 (0.724–1.602)	0.714	-	-
Hypertension (ref: none)			-	-
	1.638 (1.175–2.283)	0.004	2.283 (1.470-3.546)	0.000
BMI (ref: normal)				
Underweight	0.759 (0.409-1.406)	0.380	-	-
Overweight	0.791 (0.543-1.152)	0.221	-	-
Obese	1.003 (0.656-1.533)	0.990	-	-

## Discussion

Diabetes mellitus is a global public health challenge. This study, which sought to determine the prevalence and risk factors of DM in Ekiti State, Nigeria, revealed that the prevalence of DM among adults aged 18 years and older in Ekiti was 6.5%. We also found that DM was more prevalent among participants with post-secondary school education, employed in the formal sector, who were separated, divorced, widowed, had hypertension, and were young adults or middle-aged. Furthermore, predictors of DM were older age (33-44 and 60-69 years), higher educational level, being employed in the formal sector, and hypertension.

Our findings suggest that the prevalence of DM is high, and this may result in chronic diabetic complications such as neuropathy, retinopathy, nephropathy, coronary artery disease, stroke, and heart failure. The high prevalence of DM may also result in increased acute complications of diabetes, especially in the context of delayed health-seeking behavior, aggravated by poverty. The above will impose an additional burden on the already weak health system, which is primarily financed by out-of-pocket expenditures. And, if no urgent action is taken by policymakers and stakeholders, it may result in catastrophic expenditure, which will frustrate efforts towards the realization of the sustainable development goals (SDGs) less than a decade to the deadline [13]. Moreover, the affectation of the active working population will have a negative impact on productivity in the state.

Prevalence of diabetes mellitus

The rate of diabetes in our study is higher than the national prevalence of 5.7%, as reported in systematic reviews and meta-analyses by Uloko et al [2]. The reason for this difference may stem from the fact that the above-mentioned review included studies done over a decade ago. Similarly, the prevalence of diabetes in our study was higher than the 4.8% reported by Ogunmola et al [7]. Although their study was conducted in Ekiti State, it was done almost a decade ago and was restricted to the rural population. We suppose that the prevalence of DM may have increased as a result of nutritional transition and urbanization. Thus, the difference in the prevalence of DM between our study and the earlier studies may be attributable to the passage of time, as some authors have indicated that the prevalence of DM can double in a country within a decade [14]. Our findings are in agreement with the rising trend in global diabetes prevalence. Between 1990 and 2021, the global age-standardized prevalence of diabetes increased by more than 90%, and this increase is more than 100% in high-income nations such as North America, Europe, and the Middle East and North Africa Regions [15]. 

Some researchers reported a higher prevalence of DM than we did [16,17]. Unlike the FPG used in the index study, those researchers employed either the oral glucose tolerance test (OGTT) or glycosylated hemoglobin (HbA1c) to diagnose DM. It has been established that these three diagnostic criteria have varying degrees of sensitivity. Specifically, both OGTT and HbA1c are more sensitive than FPG in diagnosing DM [18]. Compared to our findings, studies done in south-south Nigeria revealed a higher prevalence of DM [19]. DM is more prevalent in urban areas, and since their study population was more urbanized and better developed than that of Ekiti, the higher DM rate was not unexpected. Finally, the prevalence of DM in our study was higher than the 3.6% estimate by the IDF [3]. The lower rates of DM reported by the International Diabetes Federation (IDF) may not be a true reflection of the burden of DM in Nigeria and Ekiti State.

Predictors of diabetes mellitus

In our study, the risk factors for DM were older age (33-44 and 60-69 years), higher levels of education, employment in the formal sector, and hypertension. Consistent with our findings, but with some variation, other researchers have previously reported similar findings [2,5,20,21]. All the aforementioned researchers found a positive association between older or advancing age and DM. Advancing age is associated with obesity, insulin resistance, decline in β-cell function, impaired response to incretins and lipid disorders [18]. These changes are associated with glucose dysregulation. Our study and that of others showed that hypertension was found to be associated with DM [20]. Both diseases share common pathogenic mechanisms, such as insulin resistance and inflammation, and are associated with obesity. Besides, people with hypertension are more likely to visit healthcare facilities where they could be screened for DM.

There is an association between occupation and the risk of DM. Some occupations promote physical inactivity and poor sleep, which are known risk factors for type 2 DM [22,23]. We found that those who work in the formal sector had a higher risk of developing DM compared to the self-employed and the unemployed. This may be because, in our setting, working in the formal sector is associated with extended periods of sitting, coupled with the fact that it promotes indulgence in an unhealthy and often processed diet. Furthermore, those in the formal sector may also be more educated and have better access to health facilities, which may increase their screening, especially among those who self-declared that they were already diagnosed with DM. Similar to our findings, a review determined that people with white-collar jobs had a higher risk of type 2 DM [24]. But, in contrast to our findings, some researchers reported a greater risk of DM with non-skilled workers in Asia [25]. The disparity may be due to differences in work ethics and culture.

Higher educational level was found to be an independent risk factor for DM in this study. This finding is consistent with the previously observed higher DM risk associated with employment in the formal sector. Those who had tertiary education and above are more likely to be working in the formal sector, with the attendant exposure to a diabetogenic environment. Furthermore, level of education and occupation are among the indices of socioeconomic status (SES). Thus, in our context, DM is more common among those with higher socioeconomic status. Our finding is in consonance with the previous systematic review of the relationship between the prevalence of DM and education, wealth, and BMI in LMIC [26]. In the review and systematic analysis, the prevalence of DM was found to be highest among those with the highest educational level. However, in contrast to our findings, some researchers reported an increased risk of DM among people with lower educational levels, although their studies were conducted in high-income countries [27]. This may result from the fact that the relationship between socioeconomic status and DM varies with the population studied. Generally, in high-income countries, it is more prevalent among those with low socioeconomic status, while the opposite is the case in low-income countries [28].

Our study did not reveal a gender disparity in the prevalence of DM. And reports on gender differences in the prevalence of DM have not shown a consistent pattern. While some researchers reported a higher prevalence in men, others reported no sex difference in the prevalence of DM [28,29]. Yet, age-dependent sex differences were found by some authors [30].

Unlike prior reports [21], we did not find any association between alcohol intake and DM prevalence. This may be due to the lack of quantification in our study since there is a dose-response relationship between alcohol and the risk of type 2 DM. Smoking was not associated with DM in this study. This may be due to the fact that few participants smoked. Smoking causes inflammation and insulin resistance, thereby inducing type 2 DM. Nevertheless, the relationship between diabetes and smoking is not straightforward [31].

Surprisingly, we did not find a positive association between obesity and DM prevalence. Possibly, among those who were non-obese, other risk factors for diabetes could have acted or interacted in concert to cause dysglycemia, resulting in elevated DM prevalence. This needs to be confirmed in future studies. Most researchers reported an increased risk of DM with obesity [2,21]. Due to its association with insulin resistance, obesity is a major driver of DM globally.

In our study, there was no rural-urban disparity in the prevalence of DM. A review of DM prevalence in Nigeria, as well as studies from other countries, revealed urban preponderance [2,5,32]. However, some researchers reported a higher DM prevalence in rural compared to urban areas [33], while others found no rural-urban difference in the prevalence of DM [34]. The lack of rural-urban disparity in DM prevalence in our study may be due to the adoption of a westernized diet or nutritional transition by the so-called rural dwellers. It may also be due to variations in the definition of rural and urban settlements by researchers.

The strength of this study lies in the fact that it is the first state-wide survey to determine diabetes prevalence and its risk factors in Ekiti. This survey has some limitations. Firstly, being a cross-sectional study instead of a longitudinal study, we cannot infer a causal relationship between the identified risk factors and diabetes mellitus. Secondly, the diagnosis of diabetes was based on a single fasting glucose assay. Diagnosis of DM with an oral glucose tolerance test or glycosylated hemoglobin could have detected more people. The role of family history, income, and dietary habits on the prevalence of diabetes was not reported. Additionally, the lack of quantification of alcohol in our study may limit its reported relationship with DM.

## Conclusions

The prevalence of diabetes mellitus in Ekiti State is higher than the national average. Diabetes was found to be more prevalent among those with a secondary school education or higher, those employed in the formal sector, people with raised blood pressure, and those aged 30-59 years. Compared to the never-married, DM was more prevalent among the married, and those who were separated, divorced, or widowed. The predictors of diabetes mellitus in our population include advancing age (33-44 and 60-69 years), hypertension, higher level of education, and occupation. This calls for a more aggressive public health intervention targeted at the appropriate age group, hypertensives, and the more educated people working in the formal sector.

## References

[1] Sun H, Saeedi P, Karuranga S (2023). IDF Diabetes Atlas, 10th edition. Diabetes Res Clin Pract.

[2] Uloko AE, Musa BM, Ramalan MA (2018). Prevalence and risk factors for diabetes mellitus in Nigeria: a systematic review and meta-analysis. Diabetes Ther.

[3] Alebiosu OC, Familoni OB, Ogunsemi OO (2013). Community based diabetes risk assessment in Ogun state, Nigeria (World Diabetes Foundation project 08-321). Indian J Endocrinol Metab.

[4] Avilés-Santa ML, Monroig-Rivera A, Soto-Soto A, Lindberg NM (2020). Current state of diabetes mellitus prevalence, awareness, treatment, and control in Latin America: challenges and innovative solutions to improve health outcomes across the continent. Curr Diabetes Rep.

[5] Bai A, Tao J, Tao L, Liu J (2021). Prevalence and risk factors of diabetes among adults aged 45 years or older in China: a national cross-sectional study. Endocrinol Diabetes Metab.

[6] Raimi T, Fadare J, Dada S (2020). Endocrine admissions in a tertiary hospital in Nigeria: a 5-year review of pattern and trend. Afr J Biomed Res.

[7] Ogunmola OJ, Olaifa AO, Oladapo OO, Babatunde OA (2013). Prevalence of cardiovascular risk factors among adults without obvious cardiovascular disease in a rural community in Ekiti State, Southwest Nigeria. BMC Cardiovasc Disord.

[8] (2023). National Bureau of Statistics. Population 2006-2016. https://nigerianstat.gov.ng/elibrary/read/474 .

[9] (2023). Government of Ekiti State. https://www.ekitistate.gov.ng/about-ekiti/.

[10] Oluwadiya KS Ezeani EU, Fashola A (2023). Ekiti COVID-19 and NCD Survey (EKCOVS) Protocol version 1.0. US: OSF.

[11] (2023). World Health Organization. https://www.who.int/teams/noncommunicable-diseases/surveillance/systems-tools/steps/planning-sampling.

[12] Basak A (2007). Development of a rapid and inexpensive plasma glucose estimation by two-point kinetic method based on glucose oxidase-peroxidase enzymes. Indian J Clin Biochem.

[13] Ipinnimo TM, Durowade KA (2022). Catastrophic health expenditure and impoverishment from non-communicable diseases: a comparison of private and public health facilities in Ekiti State, Southwest Nigeria. Ethiop J Health Sci.

[14] Biswas T, Tran N, Thi My Hanh H (2022). Type 2 diabetes and hypertension in Vietnam: a systematic review and meta-analysis of studies between 2000 and 2020. BMJ Open.

[15] GBD 2021 Diabetes Collaborators (2023). Global, regional, and national burden of diabetes from 1990 to 2021, with projections of prevalence to 2050: a systematic analysis for the Global Burden of Disease Study 2021. Lancet.

[16] Lawal Y, Anumah FE, Bakari AG (2018). Is glycated hemoglobin an alternative to diagnose diabetes mellitus in a Northern Nigerian population?. Ann Med Health Sci Res.

[17] Asmelash D, Asmelash Y (2019). The burden of undiagnosed diabetes mellitus in adult African population: a systematic review and meta-analysis. J Diabetes Res.

[18] Chia CW, Egan JM, Ferrucci L (2018). Age-related changes in glucose metabolism, hyperglycemia, and cardiovascular risk. Circ Res.

[19] Egbi OA, Ahmed SD (2020). Prevalence of diabetes mellitus in a rural, agrarian community in South-South Nigeria. Res J Health Sci.

[20] Adedokun AO, Ter Goon D, Owolabi EO, Adeniyi OV, Ajayi AI (2019). Prevalence, awareness, and determinants of type 2 diabetes mellitus among commercial taxi drivers in Buffalo City Metropolitan Municipality South Africa: a cross-sectional survey. Medicine (Baltimore).

[21] Liu X, Li Y, Li L (2016). Prevalence, awareness, treatment, control of type 2 diabetes mellitus and risk factors in Chinese rural population: the RuralDiab study. Sci Rep.

[22] Koh D (2018). Sedentary behaviour at work-an underappreciated occupational hazard?. Occup Med.

[23] Thivel D, Tremblay A, Genin PM, Panahi S, Rivière D, Duclos M (2018). Physical activity, inactivity, and sedentary behaviors: definitions and implications in occupational health. Front Public Health.

[24] Wu H, Meng X, Wild SH, Gasevic D, Jackson CA (2017). Socioeconomic status and prevalence of type 2 diabetes in mainland China, Hong Kong and Taiwan: a systematic review. J Global Health.

[25] Hung HH, Chan EY, Chow EY, Chung GK, Lai FT, Yeoh EK (2022). Non-skilled occupation as a risk factor of diabetes among working population: a population-based study of community-dwelling adults in Hong Kong. Health Soc Care Community.

[26] Seiglie JA, Marcus ME, Ebert C (2020). Diabetes prevalence and its relationship with education, wealth, and BMI in 29 low- and middle-income countries. Diabetes Care.

[27] Duan MF, Zhu Y, Dekker LH, Mierau JO, Corpeleijn E, Bakker SJ, Navis G (2022). Effects of education and income on incident type 2 diabetes and cardiovascular diseases: a Dutch prospective study. J Gen Intern Med.

[28] Xu Z, Yu D, Yin X, Zheng F, Li H (2017). Socioeconomic status is associated with global diabetes prevalence. Oncotarget.

[29] Nordström A, Hadrévi J, Olsson T (2016). Higher prevalence of type 2 diabetes in men than in women is associated with differences in visceral fat mass. J Clin Endocrinol Metab.

[30] Huebschmann AG, Huxley RR, Kohrt WM, Zeitler P, Regensteiner JG, Reusch JE (2019). Sex differences in the burden of type 2 diabetes and cardiovascular risk across the life course. Diabetologia.

[31] Campagna D, Alamo A, Di Pino A, Russo C, Calogero AE, Purrello F, Polosa R (2019). Smoking and diabetes: dangerous liaisons and confusing relationships. Diabetol Metab Syndr.

[32] Bavuma CM, Niyibizi JB, Bitunguhari L, Musafiri S, McQuillan R, Wild S (2022). Prevalence and characteristics associated with diabetes mellitus and impaired fasting glucose among people aged 15 to 64 years in rural and urban Rwanda: secondary data analysis of World Health Organization surveillance data. Pan Afr Med J.

[33] Tóth G, Szabó D, Sándor GL (2021). Rural-urban disparities in the prevalence of diabetes and diabetic eye complications in Hungary. Spektrum Augenheilkd.

[34] Vamos EP, Kopp MS, Keszei A (2008). Prevalence of diabetes in a large, nationally representative population sample in Hungary. Diabetes Res Clin Pract.

